# Development and validation of an Arabic vitiligo impact patient scale short form version

**DOI:** 10.1016/j.jdin.2026.01.010

**Published:** 2026-02-05

**Authors:** Mona A. Atwa, Mohammad S. Alkhowailed, Marwa Abdallah, Ahmed AlIssa, Hadeel A. Asar, Mohammed Ibrahim AlJasser, Samia Esmat, Hatim M. Alotaibi, Riham Mohyeeldeen Mohamed, Saad Altalhab, Khaled Ezzedine, Radwa El- Sayed Mahmoud Marie

**Affiliations:** a Department of Dermatology, Venereology, and Andrology. Faculty of Medicine, Suez Canal University, Ismailia, Egypt; b Department of Dermatology, College of Medicine, Qassim University, Buraidah, Qassim, Saudi Arabia; c Department of Dermatology, Venereology, and Andrology, Faculty of Medicine, Ain Shams University, Cairo, Egypt; d Derma Clinics, Riyadh, Saudi Arabia; e College of Medicine, King Saud bin Abdulaziz University for Health Sciences, Riyadh, Saudi Arabia; f King Abdullah International Medical Research Center, Riyadh, Saudi Arabia; g Division of Dermatology, Ministry of National Guard Health Affairs, Riyadh, Saudi Arabia; h Department of Dermatology, Faculty of Medicine, Cairo University, Cairo, Egypt; i Ad-Dawadmi General Hospital, Riyadh Third Health Culster, Riyadh, Saudi Arabia; j Department of Dermatology, College of Medicine, Imam Mohammad Ibn Saud Islamic University (IMSIU), Riyadh, Saudi Arabia; k Department of Dermatology, AP-HP, Henri Mondor, Créteil, France; l Université Paris-Est Créteil, EpiDermE - Epidemiology in Dermatology and Evaluation of Therapeutics, Créteil, France

**Keywords:** quality of life, scale, vitiligo

## Abstract

**Background:**

There are few tools evaluating vitiligo patients’ quality of life. A vitiligo impact patient scale short form (VIPs-SF) was developed in English.

**Objective:**

This study aims to develop a valid and reliable Arabic (AR) VIPs-SF version.

**Method:**

This cross-sectional study included 425 vitiligo patients. The VIPs-SF was translated into Arabic and was self-administered to patients according to their skin phototype. Patients also filled the AR-Dermatology Life Quality Index and the 9-question Patient Health Questionnaire for depression (PHQ-9). AR-VIPs-SF reliability, dimensional structure, and construct validity were assessed.

**Results:**

Both fair skin and dark skin AR-VIPs-SF had an excellent internal consistency (Cronbach’s α: 0.92 and 0.91) and a good test-retest reliability (intraclass correlation coefficient: 0.82 and 0.81). Confirmatory factor analysis demonstrated acceptable fit based on comparative fit index/Tucker–Lewis index/standardized root mean square residual values, although χ^2^ was significant, as expected with a large sample size. There were significant positive correlations between AR-VIPs-SF total scores and either PHQ-9 or Dermatology Life Quality Index scores (*P* < .001). Fair skin, married patients, upper limb involvement, higher Dermatology Life Quality Index, and PHQ9 scores were associated with higher VIPs-SF total score.

**Limitations:**

Absence of a discriminant validity assessment and comparison of AR-VIPs-SF to other vitiligo quality of life questionnaires. Responsiveness of AR-VIPs-SF to treatment should also be assessed.

**Conclusion:**

Arabic VIPs-SF proved valid and reliable.

**Capsule Summary**

• There are limited methods for evaluating the vitiligo burden, including the short form of the vitiligo impact patient scale.
• In this study, this short form was translated into Arabic and self-administered to 425 vitiligo patients to be statistically validated. The Arabic version was confirmed to be valid and reliable.

## Introduction

Vitiligo is a common depigmenting skin disease, with a worldwide prevalence estimated at 0.67%.^1^ The pathogenesis is complex, although it is widely accepted that melanocyte loss is caused by an autoimmune response.^2^ Psychological stresses have been associated with the onset of vitiligo.^3^ Moreover, vitiligo causes a great deal of stress in the lives of patients, and many suffer from confusion, humiliation, low self-esteem, and loneliness.^4^^,^^5^

There are only a few questionnaires that specifically address the impact of vitiligo on patients' quality of life.^6^^,^^7^ In 2015, the vitiligo impact patient scale (VIPs), which takes the skin phototype into account, was developed. The VIPs includes 29 items (19 items for all patients, 3 items specific for fair skin [FS] “phototypes I-III,” and 7 items specific for dark skin [DS] “phototypes IV-VI”).^8^

A shorter form of the VIPs has been developed. This VIPs short form (VIPs-SF) is limited to 12 items for DS and FS. The items are classified into 3 main domains: “psychological effects on daily life,” “relationships and sexuality,” and “economic constraints, care, and management of disease.” VIPs-SF offers an easier and more accurate assessment of the treatment effectiveness, whether for clinical research or routine patient care.^9^

The Dermatology Life Quality Index (DLQI) includes 10 questions designed to assess the influence of skin diseases on patients’ quality of life. It is used for people aged 16 years.^10^ The DLQI has been translated and validated into over 115 languages, including Arabic.^11^

This study aims to develop a reliable and valid Arabic VIPs-SF version to facilitate easy and accurate assessment of vitiligo burden in Arabic-speaking countries.

## Patients and methods

This cross-sectional study was performed between July 2020 and September 2022 in the Dermatology Outpatient Clinics of 5 institutions: 3 tertiary hospitals in Egypt (Ain Shams University Hospital, Suez Canal University Hospital, and Cairo University Hospital) and 2 centers in Saudi Arabia (Qassim University Medical City and National Center of Vitiligo).

Patients presenting with all clinical types of vitiligo, aged≥18 years, and of both sexes were included. Patients known to have psychiatric diseases were excluded. Sample size was calculated using the rule of thumb (N:q rule = 20:1),^12^ where N denotes the number of cases and q denotes the number of model parameters that require statistical estimates. The VIPs-SF consists of 12 items (q). Thus, the minimum sample size was 20q or *N =* 240 patients. This study included 425 vitiligo patients (185 FS and 240 DS).

The study was carried out in accordance with the STrengthening the Reporting of OBservational studies in Epidemiology (STROBE) statement items and the Helsinki Declaration principles. The study was approved by the Research Ethics Committee and Review Board, Faculty of Medicine, Suez Canal University, on October 21, 2020, with the approval number 4239. Before participating in the study, all patients signed a written informed consent.

The study authors, who are bilingual professors of dermatology and speak Arabic as their native language, translated the English VIPs-SF-FS and VIPs-SF-DS into Arabic using a multistep process that entailed a forward-backward translation. Two independent authors translated the original English questionnaire into 2 Arabic versions (A and B), and then, they reviewed these versions, made very minor editing, and adopted the final Arabic form (version C) (Supplementary File of forward translation, available on Mendeley at https://data.mendeley.com/datasets/dc53rwgnh7/1). Version C was back-translated into English by 2 other authors who were blinded to the original VIPs-SF (Supplementary File of backward translation, available on Mendeley at https://data.mendeley.com/datasets/dc53rwgnh7/1). During a cognitive interview in an online discussion forum, this back translation was validated through verifying its agreement with the original VIPs-SF. The translated versions were eventually revised, and the final Arabic forms were developed (Supplementary Files of Arabic VIPs-SF-FS, and Arabic VIPS-SF-DS, available on Mendeley at https://data.mendeley.com/datasets/dc53rwgnh7/1).

Initially, a pilot test for the Arabic VIPs-SF (AR-VIPs-SF) version was performed. The pilot study included 10% of the estimated sample size, that is, 24 patients. The questionnaire was self-administered to patients according to their skin phototype under the authors’ supervision, ensuring that patients read each item thoroughly before answering. The patients were then asked to answer a 2-point questionnaire, including a question about the items’ clarity, and another concerning any burden that was not addressed in the questionnaire. The results were analyzed by 2 authors to correct any remarks.

The final AR-VIPs-SF was administered twice to all enrolled patients, 2 weeks apart, under the authors’ supervision in a test-retest self-completed method. Patients were given the questionnaire specific to their skin phototypes and were requested to grade each item on a 6-point scale ranging from “never = 0” to “constantly = 5.” Illiterate patients were interviewed about the items. Total scores ranged from 0 to 60. At the first visit, all patients were asked to fill the Arabic validated version of DLQI and the 9-question Patient Health Questionnaire for depression (PHQ-9).^13^

### Statistical analyses

Data analysis was performed using the Statistical Package for the Social Sciences, version 25.0 (IBM Corporation), whereas the confirmatory factor analysis was conducted using Mplus software, version 7.4 (Muthén & Muthén: 1998-2017). Categorical variables were described as frequencies and percentages. Continuous variables were described as means and SDs. The Kolmogorov–Smirnov test was used to determine the normality of distributions for continuous variables.

Arabic VIPs-SF version was tested for internal consistency using Cronbach’s α. A Cronbach’s α values between 0.80 and <0.90 denote good and values ≥0.90 denote excellent internal consistency. It was tested for test-retest reliability using the intraclass correlation coefficient (ICC). ICC values between 0.75 and 0.90 denote good and values >0.90 indicate excellent reliability.

Confirmatory factor analysis was performed using a robust weighted least-squares estimator to study the factor structure of the AR-VIPs-SF. The goodness-of-fit measures were used to evaluate model fit, including comparative fit index (CFI), the goodness-of-fit index, Tucker–Lewis index (TLI), root mean squared error of approximation (RMSEA), and standardized root mean square residual (SRMR). The model fit was regarded as satisfactory when these criteria were met: TLI ≥ 0.90, CFI ≥ 0.90, and RMSEA ≤ 0.08.

Convergent validity of the AR-VIPs-SF was evaluated via Pearson’s correlation coefficients between the AR-VIPs-SF’s total and domain-specific scores with the PHQ-9 and DLQI scales’ total scores. Correlation coefficient values between 0.40 and 0.59 are moderate, 0.6 to 0.79 are strong, and 0.8 to 1.0 are very strong correlation.

The AR-VIPs-SF (FS and DS) was also tested for known-group validity using Mann–Whitney and Kruskal–Wallis tests to test differences between groups of patients with considerable depression (PHQ ≥ 10) and effects on patient’s life quality (DLQI > 10). *P* values <.05 were considered statistically significant.

Concurrent criterion validity of the AR-VIPs-SF was tested using the receiver operating characteristic curve against 2 external criteria: moderate-to-severe depression (PHQ-9 ≥ 10) and moderate-to-large DLQI (>10). Youden index-based optimal cutoff value was identified along with its area under the curve, sensitivity, specificity, positive and negative predictive values, and likelihood ratios, positive and negative.

A quantile regression model (q = 0.5) was performed to identify the predictors of the conditional median of VIPs-SF total score among the study participants. Independent variables entered in the model included variables with *P* value ≤.10 on bivariate analyses. However, the decision to exclude the variables from the model was based on their relative importance, given the percent change in model fit indices (pseudo R-squared or mean absolute error). Parameter estimates (coefficients) along with their SE and 95% CIs were used to report the model findings.

## Results

### Descriptive characteristics

This study involved 425 vitiligo patients (185 FS and 240 DS), recruited from Egyptian and Saudi populations (39.3% and 60.7%, respectively). The mean age of patients was 33.5 ± 13.2 years (range, 18-73), and females constituted 58.4%. Most patients were married (51.3%). Regarding the educational level, most patients were graduated from high school (75.1%) or less than high school (15.1%), whereas 2.8% of patients could only read and write and 7.1% of patients were illiterate. The mean age of vitiligo onset was 20.5 ± 13.7 years, and the mean disease duration was 12.9 ± 11.6 years. The most frequent body parts involved were the hands (65.6%), followed by the feet (59.5%), face (57.2%), lower limbs (56.0%), upper limbs (49.6%), trunk (42.4%), genitalia (28.5%), and neck (26.8%). About one-third of patients had a positive family history of vitiligo.

### Reliability of AR-VIPs-SF

The mean total scores of AR-VIPs-SF were 20.6 ± 14.9 and 16.5 ± 14.0 in FS and DS samples, respectively. The item score ranged from 0.72 to 2.46 in the FS sample, whereas it ranged from 0.73 to 1.78 in the DS sample, with a mean item score of 1.71 and 1.37 in the FS and DS samples, respectively. Interitem correlations ranged from 0.547 to 0.772 and 0.443 to 0.798 in FS and DS samples, respectively. AR-VIPs-SF (both FS and DS) had an excellent internal consistency (Cronbach’s α: 0.92 and 0.91, respectively). The test-retest reliability of the AR-VIPs-SF was measured in 119 FS patients and 103 DS patients who answered the VIPs questionnaires twice. Both FS and DS versions showed a good test-retest reliability (ICC: 0.82 and 0.81, respectively) (Table I).

**Table I:** Internal consistency and test-retest reliabilities of the AR-VIPs-SF

AR-VIPs-SF Scale items	Internal consistency reliability						Test-retest reliability	
	Fair skin (*n =* 185)			Dark skin (*n =* 240)			Fair skin (*n =* 119)	Dark skin (*n =* 103)
	Item mean (SD)	Corrected item-total correlation	Cronbach’s α if item deleted	Item mean (SD)	Corrected item-total correlation	Cronbach’s α if item deleted	ICC (95% CI)	ICC (95% CI)
Item 1	1.66 (1.54)	0.772	0.912	0.98 (1.37)	0.798	0.900	0.71 (0.60-0.79)	0.86 (0.79-0.90)
Item 2	1.75 (1.62)	0.718	0.914	1.43 (1.59)	0.767	0.900	0.69 (0.58-0.77)	0.80 (0.72-0.86)
Item 3	2.46 (1.74)	0.687	0.915	1.62 (1.71)	0.672	0.905	0.69 (0.57-0.77)	0.74 (0.63-0.81)
Item 4	2.03 (1.77)	0.711	0.914	1.38 (1.65)	0.760	0.901	0.61 (0.46-0.72)	0.73 (0.63-0.81)
Item 5	1.58 (1.85)	0.699	0.914	1.05 (1.58)	0.769	0.900	0.71 (0.60-0.79)	0.72 (0.62-0.80)
Item 6	1.32 (1.59)	0.703	0.914	1.48 (1.67)	0.470	0.914	0.76 (0.68-0.83)	0.69 (0.57-0.78)
Item 7	2.32 (1.78)	0.721	0.913	1.50 (1.74)	0.620	0.907	0.65 (0.52-0.74)	0.73 (0.62-0.81)
Item 8	1.17 (1.67)	0.720	0.913	0.73 (1.36)	0.602	0.908	0.74 (0.65-0.81)	0.68 (0.56-0.77)
Item 9	0.72 (1.40)	0.547	0.920	1.75 (1.79)	0.443	0.916	0.77 (0.69-0.84)	0.63 (0.50-0.73)
Item 10	1.86 (1.77)	0.567	0.920	1.78 (1.72)	0.617	0.907	0.62 (0.49-0.72)	0.68 (0.57-0.77)
Item 11	1.90 (1.80)	0.593	0.919	1.59 (1.64)	0.730	0.902	0.73 (0.63-0.80)	0.72 (0.62-0.80)
Item 12	1.78 (1.70)	0.673	0.915	1.22 (1.67)	0.635	0.906	0.70 (0.60-0.78)	0.75 (0.64-0.82)
Total scale	Cronbach’s α = 0.922			Cronbach’s α = 0.913			0.82 (0.75-0.88)	0.81 (0.74-0.87)

*AR-VIPs-SF*, Arabic vitiligo impact patient score short form; *ICC*, intraclass correlation.

### Validity of AR-VIPs-SF

#### Face validity

The AR-VIPs-SF showed high face validity. Vitiligo patients found the questions to be relevant to the impact of vitiligo on their quality of life, the language to be clear and appropriate, and the formatting to be clear and easy to read. No significant changes were suggested, indicating that the AR-VIPs-SF appears to be a good fit for its intended purpose.

#### Construct validity

##### Factor structure

**Fig 1:**
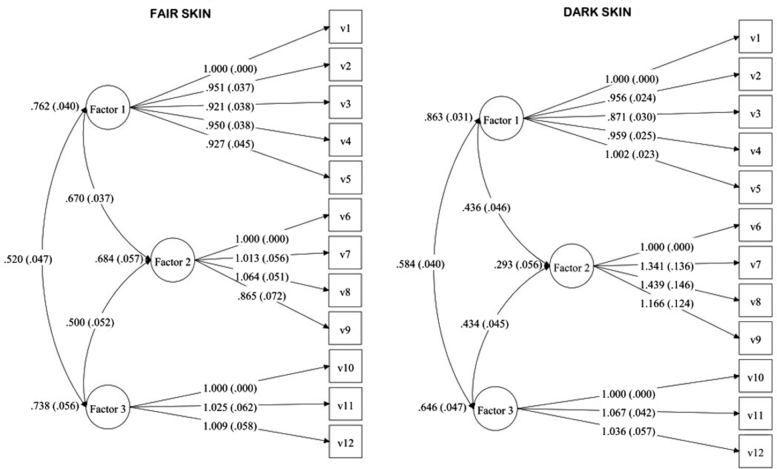
Path diagram illustrating the factor structure of the Arabic version of vitiligo impact patient score short form with item-specific factor loadings (fair skin subsample = 185 and dark skin subsample =240).

The original 3-factor model of the VIPs-SF was tested with confirmatory factor analysis in the current AR-VIPs-SF FS and DS versions, and findings are illustrated in Fig 1 and Table II.

**Table II:** Factor structure and factor loadings of the AR-VIPs-SF in vitiligo patients with fair and dark skin phototypes

AR-VIPs-SF structure		Factor loadings					
		Fair skin (*n =* 185)			Dark skin (*n* = 240)		
Factors	Items	Standard estimate	SE	*P* value	Standard estimate	SE	*P* value
Psychological effects on daily life	Item 1	0.873	0.023	.000∗	0.929	0.017	.000∗
	Item 2	0.830	0.029	.000∗	0.888	0.018	.000∗
	Item 3	0.804	0.030	.000∗	0.809	0.027	.000∗
	Item 4	0.829	0.029	.000∗	0.891	0.019	.000∗
	Item 5	0.809	0.036	.000∗	0.931	0.017	.000∗
Relationships and sexuality	Item 6	0.827	0.035	.000∗	0.541	0.052	.000∗
	Item 7	0.838	0.031	.000∗	0.726	0.041	.000∗
	Item 8	0.880	0.030	.000∗	0.778	0.045	.000∗
	Item 9	0.716	0.054	.000∗	0.631	0.045	.000∗
Economic constraints, care & management of disease							
	Item 10	0.859	0.032	.000∗	0.804	0.029	.000∗
	Item 11	0.881	0.030	.000∗	0.858	0.022	.000∗
	Item 12	0.867	0.037	.000∗	0.833	0.035	.000∗
Goodness-of-fit indices	Model fit χ^2^ (df, *P* value)	169.582 (51, .000∗)			297.976 (51, .000∗)		
	CFI	0.968			0.956		
	TLI	0.959			0.943		
	SRMR	0.059			0.082		
	RMSEA	0.112			0.142		

*AR-VIPs-SF*, Arabic vitiligo impact patient score short form; *CFI*, comparative fit index (good fit ≥0.90); *RMSEA*, root mean square error of approximation (acceptable fit ≤0.08); *SRMR*, standardized root mean square residual (good fit ≤0.08); *TLI*, Tucker–Lewis index (good if ≥0.90).

∗ Statistically significant at *P* value <.05.

Confirmatory factor analysis demonstrated acceptable fit based on CFI/TLI/SRMR values, which fell within the recommended criteria for acceptable model fit, although the chi-square in both FS and DS versions was significant (*P* < .0001), as expected with a large sample size. RMSEA estimates (FS, 0.112; 90% CI, 0.094-0.131; DS, 0.142; 90% CI, 0.127-0.158) appear elevated, indicating poor absolute model fit. This is a known limitation of RMSEA inflation in the categorical data model with moderate degrees of freedom (df = 51).^14^^,^^15^

The factor loadings for the hypothesized item-to-scale relationships were also regarded as acceptable and statistically significant (FS: range, 0.716-0.881; DS: range, 0.541-0.931).

##### Convergent validity

Significant positive strong correlations were found between the AR-VIPs-SF total scores and either PHQ-9 or DLQI scores in FS and DS samples (*P* < .001 for all). These findings indicate a good convergence with PHQ-9 and DLQI. Significant correlations were also found between all AR-VIPs-SF items and domain scores and the total PHQ-9 and DLQI scores in FS and DS samples (Table III).

**Table III:** Correlations between the total scores of the AR-VIPs-SF, PHQ-9, and DLQI in vitiligo patients with fair and dark skin phototypes

AR-VIPs-SF factors	AR-VIPs-SF item scores	Fair skin (*n =* 185)		Dark skin (*n =* 240)	
		PHQ-9	DLQI	PHQ-9	DLQI
Psychological effects on daily life	Item 1	.434∗	.575∗	.630∗	.713∗
	Item 2	.512∗	.548∗	.552∗	.666∗
	Item 3	.432∗	.479∗	.464∗	.574∗
	Item 4	.429∗	.554∗	.573∗	.639∗
	Item 5	.395∗	.656∗	.553∗	.715∗
	Total score	.531∗	.682∗	.627∗	.749∗
Relationships and sexuality economic	Item 6	.516∗	.520∗	.459∗	.374∗
	Item 7	.465∗	.538∗	.608∗	.554∗
	Item 8	.500∗	.578∗	.557∗	.621∗
	Item 9	.423∗	.607∗	.299∗	.376∗
	Total score	.593∗	.694∗	.671∗	.668∗
Constraints, care & management of disease	Item 10	.262∗	.285∗	.440∗	.459∗
	Item 11	.344∗	.345∗	.532∗	.568∗
	Item 12	.325∗	.480∗	.458∗	.539∗
	Total score	.361∗	.429∗	.562∗	.616∗
Overall VIPs-SF	Total AR-VIPs-SF score	.568∗	.695∗	.706∗	.782∗

Data presented as Pearson’s correlation coefficient (*P* value).

*AR-VIPs-SF*, Arabic vitiligo impact patient score short form; *DLQI*, Dermatology Life Quality Index; *PHQ-9*, 9-question Patient Health Questionnaire for depression.

∗ Statistically significant at *P* value <.01.

##### Known-group validity

The AR-VIPs-SF (FS version) total score was significantly associated with patient’s age and nationality, whereas the DS version total score was significantly associated with patient’s sex, education, nationality, and some body parts involved (trunk and lower limbs). Higher total scores of either FS or DS versions were significantly associated with the depression symptoms severity and the larger effect on patient’s quality of life (DLQI) (Table IV).

**Table IV:** Associations between the total score of the AR-VIPs-SF and patients’ demographic and clinical characteristics in vitiligo patients with fair and dark skin

Characteristics		Fair skin (*n =* 185)			Dark skin (*n =* 240)		
		n	Total AR-VIPs-SF score	*P* value	n	Total AR-VIPs-SF score	*P* value
Age group (y)							
<30		97	19.6 ± 14.4§	.041∗	95	17.7 ± 14.6	.226
30-39		46	20.5 ± 15.0		65	15.9 ± 12.2	
40-49		18	30.8 ± 15.4§		44	17.2 ± 14.1	
50-59		17	17.3 ± 14.3		23	14.3 ± 14.1	
60+		7	16.1 ± 12.3		13	12.0 ± 17.4	
Sex							
Female		47	17.1 ± 14.0	.072	130	13.6 ± 12.6	.000∗
Male		138	21.7 ± 15.0		110	19.9 ± 14.8	
Marital status							
Single		96	18.6 ± 15.5	.055	99	15.7 ± 13.8	.362
Married		81	22.2 ± 13.7		137	16.7 ± 13.8	
Divorced/widow		8	27.4 ± 16.6		4	29.3 ± 20.7	
Education level							
Illiterate		14	24.9 ± 19.2	.191	16 6	24.9 ± 18.6	.002∗
Read and write		6	20.5 ± 15.5		33	31.8 ± 6.4 ^c^	
Less than high school		31	25.0 ± 14.8		185	19.7 ± 16.0	
High school and above		134	19.1 ± 14.2			14.7 ± 12.7 ^c^	
Nationality							
Egyptian		99	25.4 ± 14.7	.000∗	68	22.3 ± 15.7	.000∗
Saudi		86	14.9 ± 13.0		172	14.2 ± 12.6	
Duration of vitiligo (y)							
<5		49	18.0 ± 12.6	.502	57	15.7 ± 13.2	.418
5-10		54	21.5 ± 15.1		66	15.4 ± 14.7	
More than 10		81	21.5 ± 16.0		116	17.3 ± 13.9	
Body involvement:							
Face	No Yes	72 113	20.7 ± 15.7 20.5 ± 14.4	.868	110 130	16.9 ± 15.0 16.2 ± 13.1	.977
Neck	No Yes	130 55	20.1 ± 14.4 21.7 ± 16.0	.647	181 59	15.9 ± 13.8 18.2 ± 14.5	.250
Trunk	No Yes	95 90	18.9 ± 13.1 22.3 ± 16.4	.210	150 90	14.7 ± 13.0 19.4 ± 15.1	.041∗
Upper limbs	No Yes	94 91	18.7 ± 13.3 22.5 ± 16.2	.183	120 120	15.1 ± 14.1 17.8 ± 13.8	.051
Hands	No Yes	70 115	20.9 ± 14.4 20.4 ± 15.2	.716	76 164	14.7 ± 12.7 17.3 ± 14.5	.258
Lower limbs	No Yes	86 99	20.2 ± 13.6 20.8 ± 16.0	.859	101 139	13.4 ± 13.0 18.7 ±14.2	.001∗
Feet	No Yes	79 106	19.1 ± 13.5 21.6 ± 15.8	.424	93 147	15.1 ± 13.4 17.4 ± 14.3	.175
Genitalia	No Yes	139 46	20.1 ± 14.0 21.9 ± 17.2	.845	165 75	15.6 ± 13.8 18.5 ± 14.2	.100
Family history of vitiligo							
No		125	21.0 ± 14.7	0.433	157	16.8 ± 14.3	.841
Yes		60	19.6 ± 15.3		83	15.9 ± 13.5	
Depression severity (PHQ-9)							
No or minimal		83	12.1 ± 9.6†	.000∗	143	9.6 ± 8.5†	.000∗
Mild		51	23.1 ± 13.6§		44	18.6 ± 11.7§	
Moderate		24	28.6 ± 15.6		29	29.8 ± 11.8§	
Moderately Severe		18	36.6 ± 12.8§		15	38.4 ± 11.9§	
Severe		9	30.2 ± 16.8		9	35.9 ± 15.9	
Dermatology Life Quality Index							
No effect on patient’s life		47	9.6 ± 8.4†	.000∗	88	8.0 ± 7.4†	.000∗
Small effect		66	16.4 ± 10.4†		69	11.9 ± 8.7†	
Moderate effect		39	26.6 ± 13.9		40	21.8 ± 12.0‡	
Very large		29	35.0 ± 12.5		40	35.7 ± 10.7†	
Extremely large		4	53.8 ± 4.3		3	45.3 ± 9.5	

*AR-VIPs-SF*, Arabic vitiligo impact patient score short form.

∗ Statistically significant at *P* value <0.05 (Mann–Whitney or Kruskal–Wallis tests).

† The group is significantly different from all the next groups, in post-hoc pairwise comparison (adjusted with Bonferroni correction for multiple tests).

‡ The group is significantly different from only the next group, in post-hoc pairwise comparison (adjusted with Bonferroni correction for multiple tests).

§ The group is significantly different from the first group (labeled “†”), in post-hoc pairwise comparison (adjusted with Bonferroni correction for multiple tests).

#### Criterion validity

**Fig 2:**
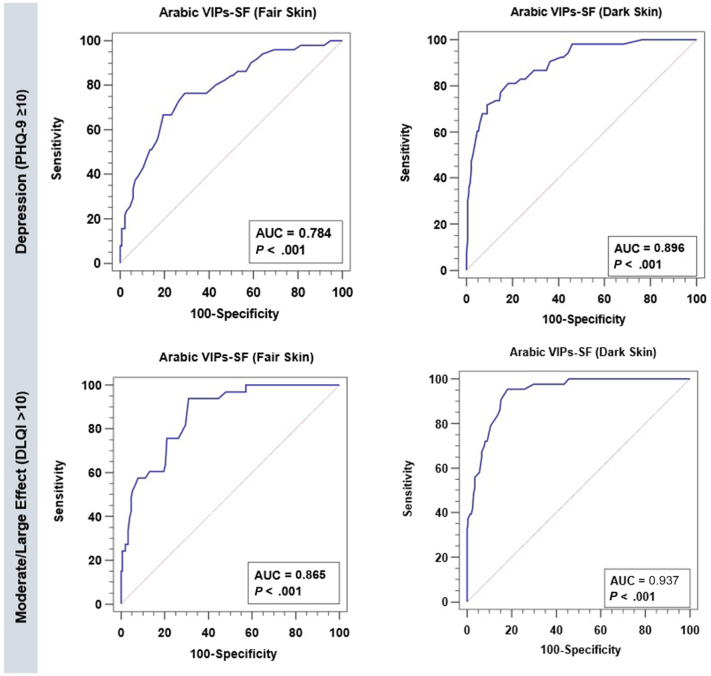
Receiver operating characteristic curve of Arabic vitiligo impact patient score short form (VIPs-SF) for depression and moderate-to-large effect on patient’s life (fair skin subsample = 185 and dark skin subsample =240). *AUC*, Area under the curve; *DLQI*, Dermatology Life Quality Index.

Youden index-based optimal cutoff values for the AR-VIPs-SF were >20 for both depression and DLQI in the FS version, and >21 for both in the DS version. The areas under the curves for either depression or DLQI criteria were statistically significant, ranging from 0.784 to 0.937, demonstrating adequate accuracy (Fig 2). Diagnostic accuracy indices are summarized in Table V and show a higher diagnostic accuracy of the AR-VIPs-SF DS version to either depression or DLQI criteria.

**Table V:** Criterion validity of the AR-VIPs-SF for depression and moderate-to-large DLQI in vitiligo patients with fair and dark skin

	Fair skin (*n =* 185)		Dark skin (*n =* 240)	
AR-VIPs-SF criterion	Depression	Mod-large DLQI	Depression	Mod-large DLQI
Youden index-based criterion	AR-VIPs-SF > 20	AR-VIPs-SF > 20	AR-VIPs-SF > 21	AR-VIPs-SF > 21
SN (95% CI)	76.5 (62.5-87.2)	93.9 (79.8-99.3)	81.1 (68.0-90.6)	95.3 (84.2-99.4)
SP (95% CI)	70.9 (62.4-78.4)	69.1 (61.1-76.3)	81.8 (75.5-87.1)	81.7 (75.6-86.9)
LR+ (95% CI)	2.63 (1.94-3.56)	3.04 (2.36-3.91)	4.46 (3.21-6.21)	5.22 (3.86-7.06)
LR− (95% CI)	0.33 (0.20-0.55)	0.09 (0.02-0.34)	0.23 (0.13-0.40)	0.06 (0.02-0.22)
PPV (95% CI)	50.0 (42.4-57.6)	39.7 (33.9-45.9)	55.8 (47.6-63.8)	53.2 (45.7-60.7)
NPV (95% CI)	88.8 (82.7-92.9)	98.1 (93.1-99.5)	93.9 (89.7-96.4)	98.8 (95.4-99.7)

*AR-VIPs-SF*, Arabic vitiligo impact patient score short form; *DLQI*, Dermatology Life Quality Index; *LR+*, positive likelihood ratio; *LR−*, negative likelihood ratio; *NPV*, negative predictive value; *PPV*, positive predictive value; *SN*, sensitivity; *SP*, specificity.

#### Predictors of VIPs-SF total score

Findings of the multiple quantile regression showed that DS was associated with 2.91 less conditional median VIPs-SF score than FS (95% CI, −5.11 to −0.71; *P* = .010). Married patients had a 3.59 higher conditional median of VIPs-SF score than unmarried patients (95% CI, 0.81-6.38; *P* = .012). Upper limbs involvement was associated with a 2.45 times higher conditional median of VIPs-SF score compared to other body sites (95% CI, 0.42-4.48; *P* = .018).

Furthermore, higher DLQI (b = 1.16; 95% CI, 0.85-1.47; *P* < .001) and PHQ9 (b = 0.97; 95% CI, 0.69-1.25; *P* < .001) total scores were associated with higher VIPs-SF total score. Compared to Egyptian patients, Saudi patients had a significantly greater positive effect of DLQI (b = 0.55; 95% CI, 0.08-1.02; *P* = .023) and a significantly less positive effect of PHQ9 (b = −0.47; 95% CI, −0.87 to −0.06; *P* = .025) on the conditional median of VIPs-SF score.

## Discussion

The VIPs-SF was developed in English. Its validation was based on correlations between its scores and the scores of the original VIPs for both FS and DS.^9^^,^^16^ However, the psychometric properties of this short form were not considered, which is a major weakness in its validation.

To our knowledge, this is the first study to develop and validate an Arabic version of the VIPs-SF, with considering its psychometric properties, to enable its application in Arabic-speaking nations.

The AR-VIPs-SF scales were revealed to be psychometrically fit, with good validity and reliability. AR-VIPs-SF versions for FS and DS had an excellent internal consistency with good test-retest reliability, which indicates good reproducibility. In the same manner, regarding the original VIPs, Cronbach’s α was 0.92 and 0.94 for FS and DS, respectively, whereas the ICC of each item was higher than 0.90 for each group and within every domain.^8^

Concerning the confirmatory factor analysis, the goodness-of-fit indices (CFI, TLI, and SRMR) provided strong evidence of good fit. RMSEA indicates poor absolute model fit due to RMSEA inflation with moderate degree of freedom in categorical models. Because current guidelines for structural equation modeling analysis emphasize interpreting model fit using multiple indices rather than RMSEA alone, the model is still considered acceptable despite RMSEA inflation.^17^ Factor loadings were also satisfactory and significant. Similar to these findings, factor analysis χ^2^ of the original VIPs was significant for both FS and DS forms with acceptable CFI and non-normed fit index.^8^

The AR-VIPs-SF for both FS and DS had a good convergence with PHQ-9 and DLQI regarding overall scores as well as item and domain scores. Regarding the concurrent criterion validity, there was a higher diagnostic accuracy of the AR-VIPs-SF DS version for either depression or DLQI criteria. Similarly, the concurrent criterion validity of the original VIPs was confirmed by significant correlations with the items of both the Short-Form-12 and the DLQI.^8^

Interestingly, this study included an evaluation of the predictors of VIPs-SF total score among enrolled patients. Married patients, FS, upper limb involvement, higher DLQI, and higher PHQ9 total scores were all associated with higher VIPs-SF total scores. These predictors were not evaluated in the original VIPs or their short forms.^8^^,^^9^

The study’s limitations included a lack of discriminant validity assessment. The interview of illiterate patients resulted in an interview bias because verbal administration might have changed the psychometric properties compared to self-administration. The cohort was derived from Egypt and Saudi Arabia. Thus, more trials are needed to determine generalizability to Arabic speakers in North Africa, Levant, and Gulf regions. Also, there is a lack of comparison between AR-VIPs-SF and the other vitiligo-specific quality of life questionnaires, such as vitiligo impact scale,^18^ Vitiligo Quality of Life instrument,^19^ and Vitiligo Life Quality Index.^20^ Furthermore, the evaluation of the AR-VIPs-SF responsiveness to treatment is recommended.

## Conclusion

The AR-VIPs-SF for FS and DS was found to be valid and reliable for assessing vitiligo burden in Arabic-speaking patients.

## Conflicts of interest

None disclosed.
